# Comparative efficacy and safety of stiripentol, cannabidiol and fenfluramine as first‐line add‐on therapies for seizures in Dravet syndrome: A network meta‐analysis

**DOI:** 10.1002/epi4.12923

**Published:** 2024-03-01

**Authors:** Renzo Guerrini, Catherine Chiron, Delphine Vandame, Warren Linley, Toby Toward

**Affiliations:** ^1^ Neuroscience Department Children's Hospital Meyer IRCCS Florence Italy; ^2^ University of Florence Florence Italy; ^3^ INSERM U1141, NeuroDiderot Université Paris Cité Paris France; ^4^ Pediatric Neurology and Reference Center for Rare Epilepsies APHP, Necker‐Enfants Malades Hospital Paris France; ^5^ Orphan Disease Division (HQ) Biocodex Gentilly France; ^6^ Paragon Market Access Ltd Chorley Lancashire UK; ^7^ Henley Health Economics Ltd Henley‐on‐Thames Oxfordshire UK

**Keywords:** cannabidiol, Dravet syndrome, fenfluramine, network meta‐analysis, stiripentol

## Abstract

**Objectives:**

Stiripentol, fenfluramine, and cannabidiol are licensed add‐on therapies to treat seizures in Dravet Syndrome (DS). There are no direct or indirect comparisons assessing their full licensed dose regimens, across different jurisdictions, as first‐line add‐on therapies in DS.

**Methods:**

We conducted a systematic review and frequentist network meta‐analysis (NMA) of randomized controlled trial (RCT) data for licensed add‐on DS therapies. We compared the proportions of patients experiencing: reductions from baseline in monthly convulsive seizure frequency (MCSF) of ≥50% (clinically meaningful), ≥75% (profound), and 100% (seizure‐free); serious adverse events (SAEs); discontinuations due to AEs.

**Results:**

We identified relevant data from two placebo‐controlled RCTs for each drug. Stiripentol 50 mg/kg/day and fenfluramine 0.7 mg/kg/day had similar efficacy in achieving ≥50% (clinically meaningful) and ≥75% (profound) reductions from baseline in MCSF (absolute risk difference [RD] for stiripentol versus fenfluramine 1% [95% confidence interval: −20% to 22%; *p* = 0.93] and 6% [−15% to 27%; *p* = 0.59], respectively), and both were statistically superior (*p* < 0.05) to licensed dose regimens of cannabidiol (10 or 20 mg/kg/day, with/irrespective of clobazam) for these outcomes. Stiripentol was statistically superior in achieving seizure‐free intervals compared to fenfluramine (RD = 26% [CI: 8% to 44%; *p* < 0.01]) and licensed dose regimens of cannabidiol. There were no significant differences in the proportions of patients experiencing SAEs. The risk of discontinuations due to AEs was lower for stiripentol, although the stiripentol trials were shorter.

**Significance:**

This NMA of RCT data indicates stiripentol, as a first‐line add‐on therapy in DS, is at least as effective as fenfluramine and both are more effective than cannabidiol in reducing convulsive seizures. No significant difference in the incidence of SAEs between the three add‐on agents was observed, but stiripentol may have a lower risk of discontinuations due to AEs. These results may inform clinical decision‐making and the continued development of guidelines for the treatment of people with DS.

**Plain Language Summary:**

This study compared three drugs (stiripentol, fenfluramine, and cannabidiol) used alongside other medications for managing seizures in a severe type of epilepsy called DS. The study found that stiripentol and fenfluramine were similarly effective in reducing seizures and both were more effective than cannabidiol. Stiripentol was the best drug for stopping seizures completely based on the available clinical trial data. All three drugs had similar rates of serious side effects, but stiripentol had a lower chance of being stopped due to side effects. This information can help guide treatment choices for people with DS.

**Key Point**

- Stiripentol, fenfluramine, and cannabidiol are licensed internationally as add‐on therapies for managing seizures in Dravet syndrome (DS).
- No comparative trials exist. Prior indirect treatment comparisons (ITCs) do not account for their dose regimens in different countries.
- This ITC shows stiripentol is at least as effective as fenfluramine in reducing seizures in DS. Both are superior to cannabidiol regimens.
- No major differences in serious adverse events between therapies were noted, but fewer patients stopped stiripentol due to adverse events.
- Findings support international consensus‐based treatment recommendations favoring stiripentol and fenfluramine over cannabidiol in DS.

## 1 INTRODUCTION

Dravet syndrome (DS, previously known as severe myoclonic epilepsy in infancy) is a rare and severe developmental and epileptic encephalopathy.^1^, ^2^ It is characterized by frequent, convulsive seizures arising in the first year of life, followed by developmental delay and cognitive impairment, which impair patient and carer quality of life.^3^, ^4^ Around 15%–20% of children with DS die before reaching adulthood primarily due to status epilepticus (SE), and sudden unexpected death in epilepsy (SUDEP).^5^, ^6^

In DS, high convulsive seizure frequency is associated with an increased risk of death and developmental comorbidities and contributes to impaired quality of life.^7^, ^8^ Reducing convulsive seizure frequency is, therefore, a key goal of treatment. Treatment recommendations suggest initiating anti‐seizure medication (ASM) with valproate or valproate and clobazam^9^, ^10^, ^11^; however, as most patients' seizures are inadequately controlled with these treatments, additional add‐on therapy is typically required.^9^, ^10^

Stiripentol (Diacomit®),^12^, ^13^ fenfluramine (Fintepla®),^14^, ^15^ and pharmaceutical‐grade cannabidiol (Epidiolex®/Epidyolex®)^16^, ^17^ are licensed specifically as add‐on ASMs for DS. Stiripentol was first licensed in Europe in 2007, where in some countries it is considered a part of standard care.^11^ It was subsequently licensed in other jurisdictions including Canada, Japan, and, in 2018, in the USA. Of note, the stiripentol license in Europe requires concomitant use of valproate and clobazam^13^ but in the USA the license only stipulates clobazam.^12^ Fenfluramine and cannabidiol were licensed in Europe and the USA between 2018 and 2019. The cannabidiol license in Europe requires concomitant use of clobazam,^17^ but there are no stipulations for clobazam use in the USA license.^16^

All three add‐on therapies were licensed on the basis of placebo‐controlled randomized controlled trials (RCTs)^18^, ^19^, ^20^, ^21^, ^22^, ^23^ and there are no direct comparative trials of their relative effects. However, a recent international consensus paper on the diagnosis and management of DS positions stiripentol and fenfluramine ahead of cannabidiol in the treatment pathway.^9^ We conducted a study to indirectly compare the efficacy and safety of stiripentol, fenfluramine, and cannabidiol when used as initial (first‐line) add‐on therapies across their licensed dose regimens in DS. Given the availability of multiple trials of each intervention, we applied network meta‐analysis (NMA), which is an indirect comparison method accepted by health technology assessment (HTA) agencies and guideline developers around the world.^24^

## 2 METHODS

### 2.1 Systematic searches and study selection

We searched for published randomized controlled trial (RCT) data for stiripentol, fenfluramine, and cannabidiol at their DS‐licensed doses in PubMed and Embase® using a Cochrane sensitivity and precision maximizing search filter for RCTs,^25^ combined with appropriate terms and subject headings for DS (see Table S1). We also searched the Cochrane Central Register of Controlled Trials (CENTRAL), the Cochrane Database of Systematic Reviews, and European and US regulatory authority and major HTA websites. Major epilepsy conference proceedings were searched for abstracts using free text terms for DS. Database searches were initially conducted from inception up to 12 December 2022 and then updated to 30 June 2023 (PROSPERO registration: CRD42023444136).

Full eligibility criteria for study selection are reported in Table S2. Screening was conducted by two reviewers, and data extraction was performed by one reviewer and validated by the second. The primary efficacy outcome of interest was the proportion of patients achieving ≥50% (clinically meaningful) reduction from baseline in monthly convulsive seizure frequency (MCSF), with analyses also planned for reductions ≥75% (profound) and 100% (seizure‐free). Convulsive seizures were the focus of efficacy as they are the key efficacy endpoint used for the licensing of add‐on therapies,^12^, ^13^, ^14^, ^15^, ^16^, ^17^ and are associated with adverse near‐ to long‐term health outcomes for patients, including seizure‐related mortality.^26^, ^27^ Standardized safety outcomes that would have a clear impact on patient health, quality of life, treatment continuation, or health care resource use, were the focus of interest: the incidence of any serious AEs (SAEs, usually defined as resulting in death, or life‐threatening, or requiring hospitalization, or resulting in persistent or significant disability or incapacity, or leading to congenital anomaly^28^) and discontinuations due to AEs.

The feasibility of undertaking indirect treatment comparisons was assessed by comparing study designs, eligibility criteria, study endpoints, and baseline characteristics of enrolled participants. Study quality was assessed using the Cochrane Risk of Bias 2 tool (ROB2).^29^

### 2.2 Data analysis

Frequentist NMAs were conducted using MetaInsight v4.1.0 (April 2023), employing the netmeta package in R statistical software.^30^ Random effects models to account for study heterogeneity were used, and two‐sided *p*‐values for indirect estimates of relative treatment effect estimates were calculated using the methods described by Altman 2011.^31^ Fixed effect models were explored in sensitivity analyses.

Where data allowed, analyses were conducted to reflect the different licensing requirements for concomitant therapy for each of the add‐on therapies across different jurisdictions. Relative treatment effects in the NMA were assessed using relative risks (RR) where possible. As RR cannot be estimated in cases where there are zero events, absolute risk differences (RD), which can accommodate zero cell counts,^32^ were also estimated to enable robust comparative treatment effect estimates for all outcomes. This approach enabled the use of the largest possible dataset and ensured consistency in the presentation of the comparative treatment effect estimates. From RD estimates it is also possible to calculate the number needed to treat (NNT) with each intervention (added on to standard of care therapy) for one more patient to achieve the outcome of interest compared with placebo (added on to standard of care therapy): NNT = 1/|RD|.^32^ NNTs were therefore calculated for pairwise comparisons versus placebo for RD that were statistically significant to facilitate a clinical interpretation of the NMA results.

## 3 RESULTS

### 3.1 Systematic search results

The literature search identified two RCTs for stiripentol (STICLO‐France^18^ and STICLO‐Italy^19^), three RCTs for fenfluramine (Study 1,^20^ Study 2 [previously known as Study 1504]^21^ and Study 3^33^), and three RCTs for cannabidiol (GWPCARE1 Part A,^34^ GWPCARE 1 Part B,^22^ GWPCARE2^23^). Other publications (e.g., poster and abstract publications) and reports (e.g., regulatory and HTA reports) associated with the RCTs were also identified (see PRISMA diagram in Figure S1).

Based on feasibility and quality assessments, the stiripentol, fenfluramine, and cannabidiol RCTs were judged to be sufficiently similar in their designs, efficacy, and safety endpoints (Table S3) and baseline characteristics (Table S4), and to be of sufficiently low risk of bias (Figure S2), to permit robust NMAs using data from:

- STICLO‐France^18^ and STICLO‐Italy^19^ for stiripentol 50 mg/kg/day, supplemented with data from published regulatory reports on SAEs and discontinuations due to AEs,^35^ and recent re‐analyses of the STICLO RCTs for 75% responder rates (^36^ manufacturer data on file).
- Study 1^20^ and Study 3^33^ for fenfluramine 0.7 mg/kg/day.
- GWPCARE 1 (part B)^22^ and GWPCARE 2^23^ for cannabidiol 10 and 20 mg/kg/day, irrespective of the use of concomitant clobazam (per cannabidiol's licensed indication in the USA).^16^
- Subgroup analyses of GWPCARE 1 (part B) and GWPCARE 2 for cannabidiol 10 and 20 mg/kg/day in combination with clobazam^17^, ^37^, ^38^ (per cannabidiol's licensed indication in Europe^17^).

**FIGURE 1:**
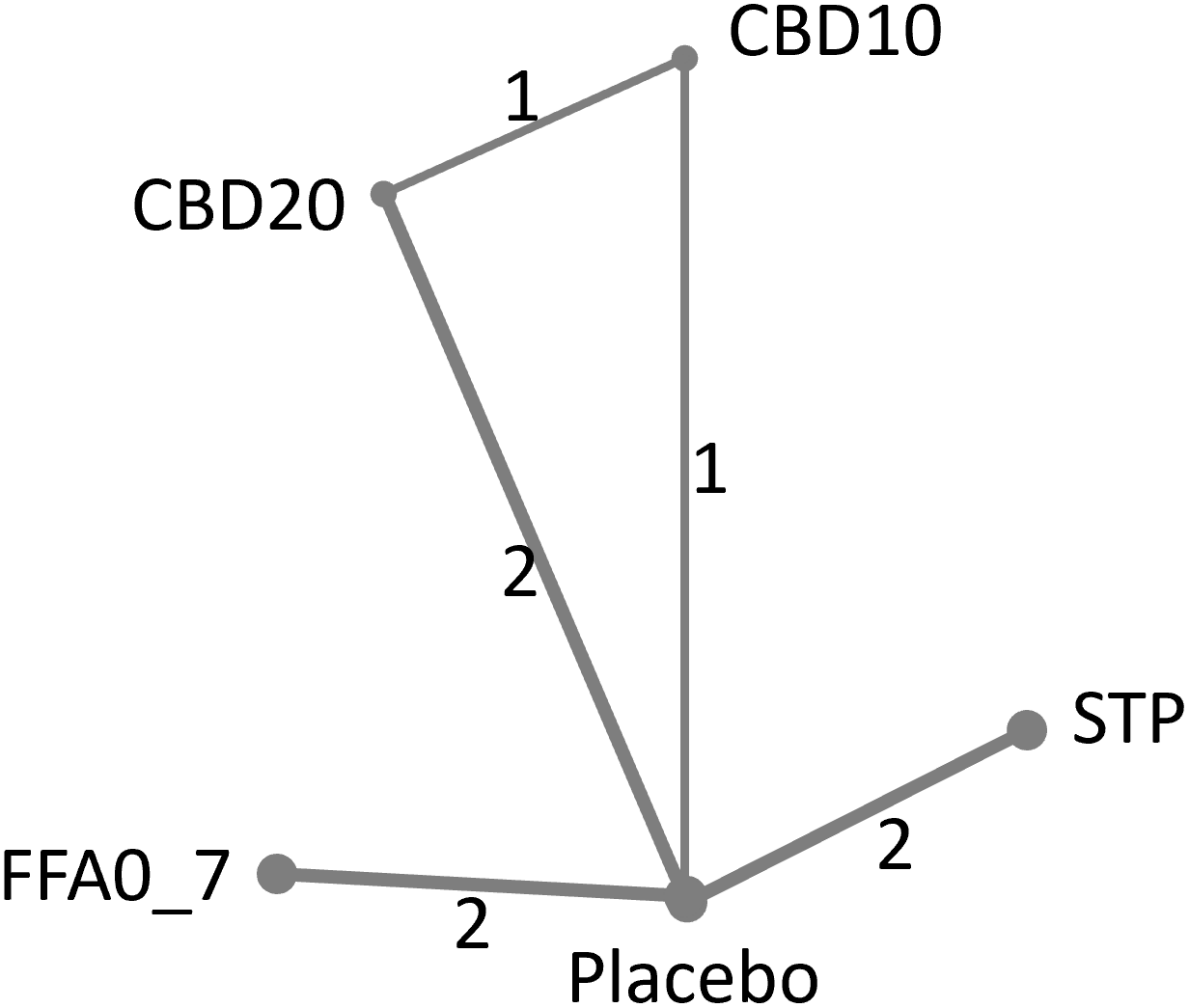
Trial network for all outcomes. CBD10, cannabidiol 10 mg/kg/day; CBD20, cannabidiol 20 mg/kg/day; FFA0_7, fenfluramine 0.7 mg/kg/day; STP, stiripentol 50 mg/kg/day. Numbers on lines depict number of RCTs providing direct comparisons between treatments.

The network of trials used in the NMAs is presented in Figure 1. The efficacy and safety data used in the NMAs are provided in Table 1. Although the stiripentol license in the USA does not specify the use of concomitant valproate,^12^ there are no published RCT data available to conduct analyses for stiripentol without concomitant valproate. Fenfluramine Study 2 was excluded from the NMAs on the basis that all patients enrolled in that trial were taking concomitant stiripentol,^21^ and it is not clinically logical to include data from this study of fenfluramine plus stiripentol in a comparison against stiripentol, particularly in the context of first‐line add‐on use. In addition, the fenfluramine 0.2 mg/kg/day arm of Study 1 and Study 3 was excluded from the NMAs as this is an initiation dose and not the target maintenance dose of fenfluramine.^15^ The cannabidiol study GWPCARE1 Part A^34^ was excluded as it did not report outcomes of interest.

**TABLE 1:** Data used in NMAs.

RCT	Treatment arm	*n*	Responder rates (reduction from baseline in MCSF)			Serious AEs *n*/*N* (%)	Discontinuations due to AEs
			≥50% *n*/*N* (%)	≥75% *n*/*N* (%)	100% *n*/*N* (%)		
*Stiripentol RCTs*							
STICLO‐France^18^, ^52^	Stiripentol 50 mg/kg/day	21	15/21 (71.5)	12/21 (57.1)	9/21 (42.9)	2/21 (9.5)	1/21 (4.8)
	Placebo	20	1/20 (5)	0/20 (0)	0/20 (0)	3/20 (15)	4/20 (20)
STICLO‐Italy^19^, ^52^	Stiripentol 50 mg/kg/day	12	8/12 (66.7)	6/12 (50)	3/12 (25)	0/12 (0)	1/12 (8.3)
	Placebo	11	1/11 (9.1)	1/11 (9.1)	0/11 (0)	0/11 (0)	2/11 (18.2)
*Fenfluramine RCTs*							
Study 1^20^	FFA 0.7 mg/kg/day (max 26 mg/day)	40	27/40 (67.5)	20/40 (50.0)	3/40 (7.5)	5/40 (12.5)	5/40 (12.5)
	Placebo	40	5/40 (12.5)	1/40 (2.5)	0/40 (0)	4/40 (10.0)	0/40 (0)
Study 3^33^	FFA 0.7 mg/kg/day (max 26 mg/day)	49	35/49 (71.4)	23/49 (46.9)	6/49 (12.2)	3/49 (6.1)	2/49 (4.1)
	Placebo	48	3/48 (6.3)	2/48 (4.2)	0/48 (0)	2/48 (4.2)	1/48 (2.1)
*Cannabidiol RCTs*							
GWPCARE1B^22^	CBD 20 mg/kg/day	61	26/61 (42.6)	14/61 (23.0)	3/61 (4.9)	10/61 (16.4)	8/61 (13.1)
	Placebo	59	16/59 (27.1)	7/59 (11.9)	0/59 (0)	3/59 (5.1)	1/59 (1.7)
GWPCARE2^23^	CBD 10 mg/kg/day	66	29/66 (43.9)	20/66 (30.3)	2/66 (3.0)	13/64 (20.3)	0/64 (0)
	CBD 20 mg/kg/day	67	33/67 (49.3)	12/67 (17.9)	3/67 (4.5)	17/69 (24.6)	5/69 (7.2)
	Placebo	65	17/65 (26.2)	4/65 (6.2)	1/65 (1.5)	10/65 (15.4)	0/65 (0)
*Cannabidiol RCTs—subgroup taking clobazam*							
GWPCARE1B^37^, ^38^	CBD 20 mg/kg/day	40	19/40 (47.5)	10/40 (25.0)	3/40 (7.5)	8/40 (20.0)	6/40 (15)
	Placebo	38	9/38 (23.7)	5/38 (13.2)	0/38 (0)	1/38 (2.6)	1/38 (2.6)
GWPCARE2^37^, ^38^	CBD 10 mg/kg/day	45	25/45 (55.6)	16/45 (35.6)	2/45 (4.4)	10/44 (22.7)	0/44 (0)
	CBD 20 mg/kg/day	40	25/40 (62.5)	10/40 (25.0)	2/40 (5)	11/41 (26.8)	4/41 (9.8)
	Placebo	41	15/41 (36.6)	4/41 (9.8)	1/41 (2.4)	7/41 (17.1)	0/41 (0)

Abbreviations: AE, adverse events; MCSF, monthly convulsive seizure frequency; RCTs, randomized controlled trials.

### 3.2 Efficacy endpoints

#### 3.2.1 ≥50% reduction in monthly convulsive seizure frequency

The pairwise RR for achieving ≥50% (i.e., a clinically meaningful) reduction from baseline in MCSF versus placebo is presented in Figure S3. Stiripentol, fenfluramine, and cannabidiol were all statistically significantly superior to placebo. Stiripentol was numerically the most effective of the three drugs in the analyses (RR: 10.20; 95% CI 2.62 to 39.66), followed by fenfluramine (RR: 7.20; 95% CI 3.67 to 14.11), cannabidiol 20 mg/kg/day (RR: 1.73; 95% CI 1.22 to 2.45 in the full trial population, and RR: 1.80; 95% CI 1.23 to 2.64 in the subgroup taking clobazam), and cannabidiol 10 mg/kg/day (RR: 1.58; 95% CI 1.03 to 2.42 in the full trial population and RR: 1.58; 95% CI 1.02 to 2.44 in the subgroup taking clobazam).

**FIGURE 2:**
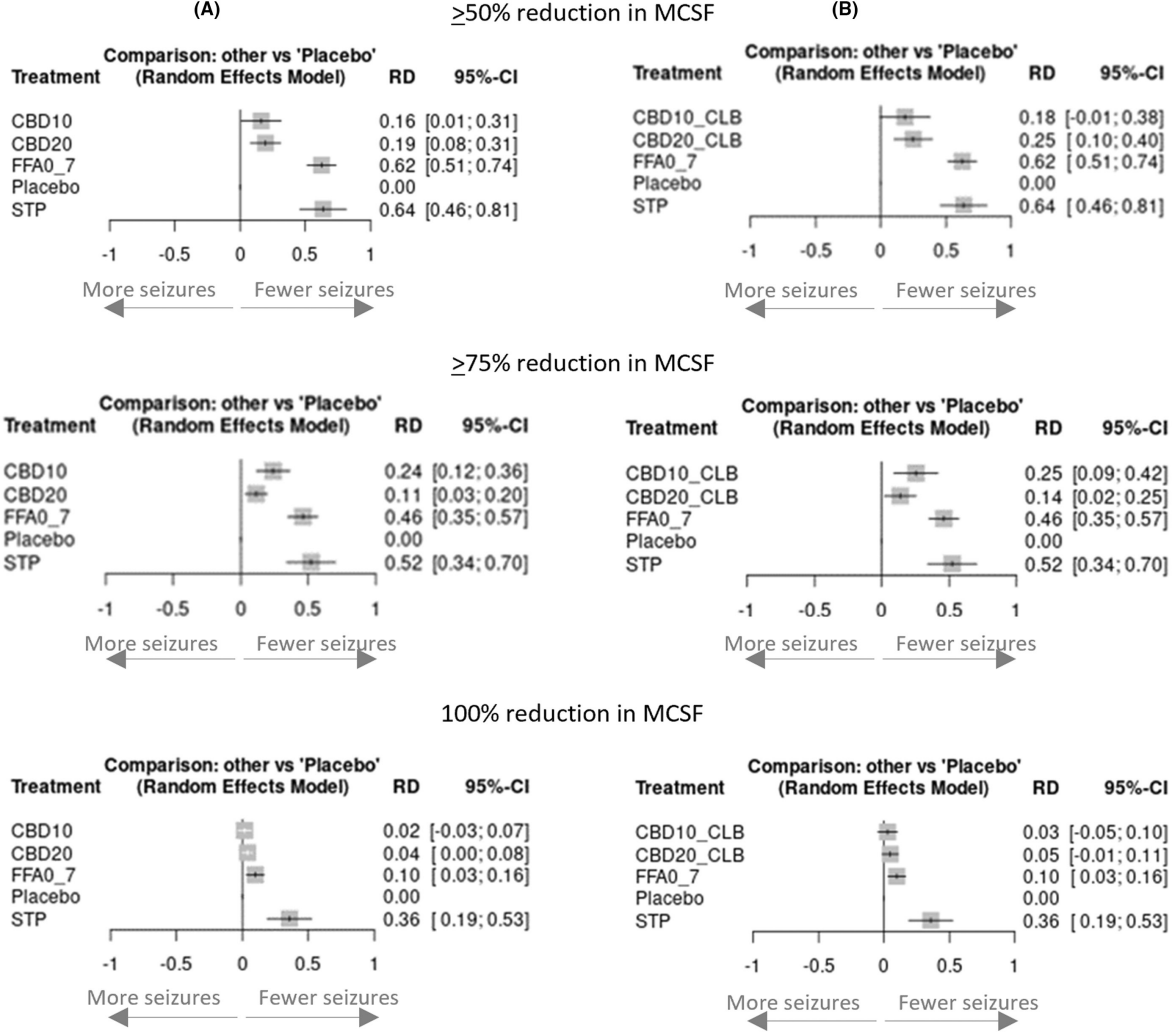
Pairwise risk differences versus placebo for achieving ≥50%, ≥75% and 100% reductions in MCSF. (A) Responder rates using full cannabidiol trial populations; (B) Responder rates using subgroup of cannabidiol trial populations taking clobazam. CBD10, cannabidiol 10 mg/kg/day; CBD10_CLB, cannabidiol 10 mg/kg/day + clobazam; CBD20, cannabidiol 20 mg/kg/day; CBD20_CLB, cannabidiol 20 mg/kg/day + clobazam; FFA0_7, fenfluramine 0.7 mg/kg/day; MCSF, monthly convulsive seizure frequency; RD, risk difference; STP, stiripentol 50 mg/kg/day; 95%‐CI, 95% confidence intervals.

Based on RD (Figure 2), stiripentol was numerically the most effective of the three drugs, with a RD versus placebo of 64% and NNT of 2, followed by fenfluramine (RD: 62%; NNT: 2), cannabidiol 20 mg/kg/day (RD: 19%; NNT: 6 in the full trial population, and RD: 25%; NNT: 4 in the subgroup taking clobazam) and cannabidiol 10 mg/kg/day (RD: 16%; NNT: 7 in the full trial population, and non‐significant RD: 18% in the subgroup taking clobazam). Indirect estimates of RDs indicated that stiripentol and fenfluramine were statistically significantly superior to licensed dose regimens of cannabidiol used with or irrespective of concomitant clobazam (Table 2).

**TABLE 2:** Indirect comparisons of ≥50%, ≥75%, and 100% reductions from baseline in MCSF for stiripentol versus fenfluramine versus cannabidiol.

Indirect comparisons—Risk difference [95% confidence interval] for 50% responder rate								
A—using full cannabidiol trial populations								
	STP	*p*‐value	FFA0_7	*p*‐value	CBD20	*p*‐value	CBD10	*p*‐value
STP	STP							
FFA0_7	0.01 [−0.20; 0.22]	0.93	FFA0_7					
CBD20	**0.44 [0.23; 0.65]**	**<0.0001**	**0.43 [0.27; 0.59]**	**<0.0001**	CBD20			
CBD10	**0.47 [0.24; 0.71]**	**0.0001**	**0.46 [0.27; 0.65]**	**<0.0001**	0.03 [−0.12; 0.19]	0.72	CBD10	
Placebo	**0.64 [0.46; 0.81]**	**<0.0001**	**0.62 [0.51; 0.74]**	**<0.0001**	**0.19 [0.08; 0.31]**	**0.0013**	**0.16 [0.01; 0.31]**	**0.036**

B—using subgroup of cannabidiol trial populations taking clobazam								
	STP	*p*‐value	FFA0_7	*p*‐value	CBD20_CLB	*p*‐value	CBD10_CLB	*p*‐value
STP	STP							
FFA0_7	0.01 [−0.20; 0.22]	0.93	FFA0_7					
CBD20_CLB	**0.39 [0.16; 0.62]**	**0.0009**	**0.38 [0.19; 0.56]**	**<0.0001**	CBD20_CLB			
CBD10_CLB	**0.45 [0.19; 0.71]**	**0.0007**	**0.44 [0.22; 0.66]**	**0.0001**	0.06 [−0.13; 0.26]	0.56	CBD10_CLB	
Placebo	**0.64 [0.46; 0.81]**	**<0.0001**	**0.62 [0.51; 0.74]**	**<0.0001**	**0.25 [0.10; 0.40]**	**0.0011**	0.18 [−0.01; 0.38]	0.070

Indirect comparisons—Risk Difference [95% Confidence interval] for 75% responder rate								
A—using full cannabidiol trial populations								
	STP	*p*‐value	FFA0_7	*p*‐value	CBD10	*p*‐value	CBD20	*p*‐value
STP	STP							
FFA0_7	0.06 [−0.15; 0.27]	0.59	FFA0_7					
CBD10	**0.28 [0.06; 0.50]**	**0.0126**	**0.22 [0.05; 0.39]**	**0.0112**	CBD10			
CBD20	**0.41 [0.21; 0.61]**	**<0.0001**	**0.35 [0.21; 0.48]**	**<0.0001**	0.13 [−0.01; 0.26]	0.0587	CBD20	
Placebo	**0.52 [0.34; 0.70]**	**<0.0001**	**0.46 [0.35; 0.57]**	**<0.0001**	**0.24 [0.12; 0.36]**	**0.0001**	**0.11 [0.03; 0.20]**	**0.0112**

B—using subgroup of cannabidiol trial populations taking clobazam								
	STP	*p*‐value	FFA0_7	*p*‐value	CBD10_CLB	*p*‐value	CBD20_CLB	*p*‐value
STP	STP							
FFA0_7	0.06 [−0.15; 0.27]	0.59	FFA0_7					
CBD10_CLB	**0.27 [0.03; 0.51]**	**0.0272**	**0.21 [0.01; 0.40]**	**0.0345**	CBD10_CLB			
CBD20_CLB	**0.39 [0.17; 0.60]**	**0.0004**	**0.32 [0.16; 0.49]**	**0.0002**	0.12 [−0.06; 0.29]	0.18	CBD20_CLB	
Placebo	**0.52 [0.34; 0.70]**	**<0.0001**	**0.46 [0.35; 0.57]**	**<0.0001**	**0.25 [0.09; 0.42]**	**0.0030**	**0.14 [0.02; 0.25**]	**0.0169**

Indirect comparisons—Risk Difference [95% Confidence interval] for 100% responder rate								
A—using full cannabidiol trial populations								
	STP	p‐value	FFA0_7	*p*‐value	CBD20	*p*‐value	CBD10	*p*‐value
STP	STP							
FFA0_7	**0.26 [0.08; 0.44]**	**0.0047**	FFA0_7					
CBD20	**0.32 [0.15; 0.49]**	**0.0003**	0.06 [−0.02; 0.14]	0.14	CBD20			
CBD10	**0.34 [0.16; 0.52]**	**0.0002**	**0.08 [0.00; 0.16]**	**0.0496**	0.02 [−0.04; 0.08]	0.52	CBD10	
Placebo	**0.36 [0.19; 0.53]**	**<0.0001**	**0.10 [0.03; 0.16]**	**0.0026**	**0.04 [0.00; 0.08]**	**0.0496**	0.02 [−0.03; 0.07]	0.44

B—using subgroup of cannabidiol trial populations taking clobazam								
	STP	*p*‐value	FFA0_7	*p*‐value	CBD20_CLB	*p*‐value	CBD10_CLB	*p*‐value
STP	STP							
FFA0_7	**0.26 [0.08; 0.44]**	**0.0047**	FFA0_7					
CBD20_CLB	**0.31 [0.13; 0.49]**	**0.0008**	0.05 [−0.04; 0.14]	0.28	CBD20_CLB			
CBD10_CLB	**0.33 [0.15; 0.51]**	**0.0004**	0.07 [−0.03; 0.17]	0.17	0.02 [−0.06; 0.10]	0.64	CBD10_CLB	
Placebo	**0.36 [0.19; 0.53]**	**<0.0001**	**0.10 [0.03; 0.16]**	**0.00262**	0.05 [−0.01; 0.11]	0.10	0.03 [−0.05; 0.10]	0.44

*Note*: Treatments are ranked from best to worst along the leading diagonal. Indirect estimates of risk differences presented for treatments in columns versus treatments in rows. Bold figures are statistically significant (*p*‐value ≤0.05).

Abbreviations: CBD10, cannabidiol 10 mg/kg/day; CBD10_CLB, cannabidiol 10 mg/kg/day + clobazam; CBD20, cannabidiol 20 mg/kg/day; CBD20_CLB, cannabidiol 20 mg/kg/day + clobazam; FFA0_7, fenfluramine 0.7 mg/kg/day; STP, stiripentol 50 mg/kg/day.

#### 3.2.2 ≥75% and 100% reduction in monthly convulsive seizure frequency

Due to zero event rates in one or more arms of the RCTs, RR could not be estimated for achieving ≥75% and 100% reductions from baseline in MCSF for all interventions.

Based on RD for a ≥75% (i.e., profound) reduction from baseline in MCSF (Figure 2; Table 2), stiripentol was numerically the most effective of the three drugs, with a RD versus placebo of 52% and NNT of 2; followed by fenfluramine (RD: 46%; NNT: 3); cannabidiol 10 mg/kg/day (RD: 24%; NNT: 5 in the full trial population, and RD: 25%; NNT: 4 in the subgroup taking clobazam); and cannabidiol 20 mg/kg/day (RD: 11%; NNT: 10 in the full trial population, and RD: 14%; NNT: 8 in the subgroup taking clobazam). Indirect estimates of RDs indicated that stiripentol and fenfluramine were statistically significantly superior to licensed dose regimens of cannabidiol used with or irrespective of concomitant clobazam use (Table 2).

For 100% reduction from baseline in MCSF (i.e., seizure‐free; Figure 2; Table 2), stiripentol was the most effective of the three interventions, with a RD versus placebo of 36% and NNT of 3; followed by fenfluramine (RD: 10%; NNT: 10); cannabidiol 20 mg/kg/day (RD: 4%, NNT: 25 in the full trial population, and non‐significant RD: 5% in the subgroup taking clobazam); and cannabidiol 10 mg/kg/day (non‐significant RD: 2% in the full trial population, and non‐significant RD: 3% in the subgroup taking clobazam). Indirect estimates of RDs indicated that stiripentol was statistically superior to fenfluramine and licensed dose regimens of cannabidiol, with or irrespective of concomitant clobazam use, for this endpoint (Table 2).

### 3.3 Safety endpoints

#### 3.3.1 Serious adverse event rates

**FIGURE 3:**
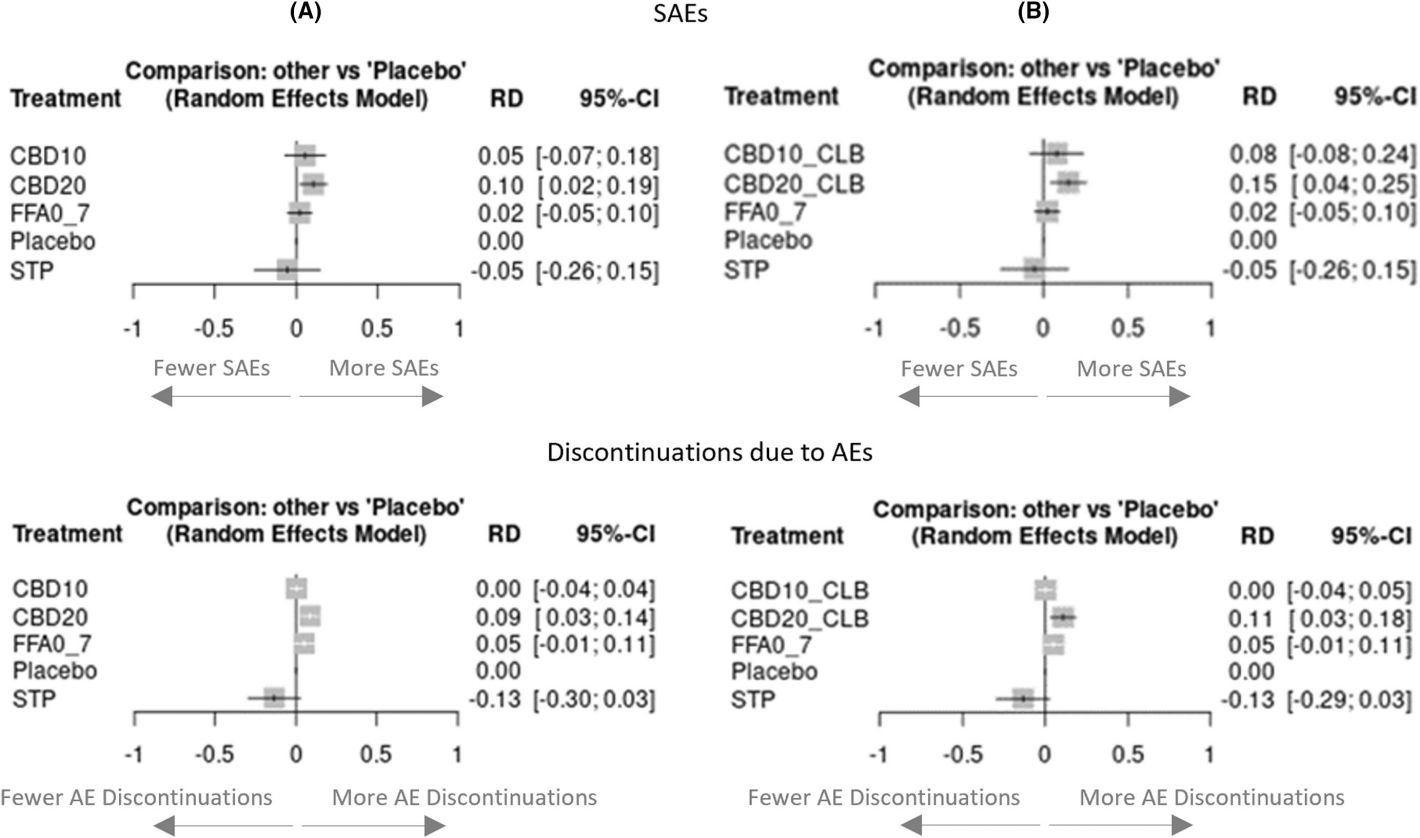
Pairwise risk differences versus placebo for SAEs and Discontinuations due to AEs. (A) Risk differences using full cannabidiol trial populations; (B) Risk differences using subgroup of cannabidiol trial populations taking clobazam. AEs, adverse events; CBD10, cannabidiol 10 mg/kg/day; CBD10_CLB, cannabidiol 10 mg/kg/day + clobazam; CBD20, cannabidiol 20 mg/kg/day; CBD20_CLB, cannabidiol 20 mg/kg/day + clobazam; FFA0_7, fenfluramine 0.7 mg/kg/day; RD, risk difference; SAEs, serious adverse events; STP, stiripentol 50 mg/kg/day; 95% CI, 95% confidence intervals.

The pairwise RRs for patients experiencing SAEs are presented in Figure S4. There was no significant difference compared with placebo in the incidence of patients experiencing SAEs with stiripentol, fenfluramine, or cannabidiol 10 mg/kg/day. A significantly increased risk of SAEs was only observed with cannabidiol 20 mg/kg/day in the full trial population (RR: 1.90; 95% CI 1.03 to 3.50). Patients taking stiripentol were numerically the least likely to experience SAEs, with a lower point estimate than placebo (RR: 0.63; 95% CI 0.12 to 3.41). Results were similar when using RDs, with the patient incidence of SAEs significantly greater with cannabidiol 20 mg/kg/day in both the full cannabidiol trial populations and the subgroup taking clobazam (Figure 3). There was no significant difference in the patient incidence of SAEs between stiripentol, fenfluramine, and any of the licensed cannabidiol dose regimens (see Table S5).

#### 3.3.2 Discontinuations due to adverse events

Due to zero events in one or more treatment arms, RRs could not be estimated for discontinuations due to AEs for all interventions. Based on RDs, there were no significant differences compared with placebo in the incidence of patient discontinuations due to AEs with stiripentol, fenfluramine, or cannabidiol; the risk was numerically lowest with stiripentol (Figure 3). In indirect comparisons of RDs, the risk of discontinuations due to AEs with stiripentol was statistically significantly (*p* < 0.05) lower for stiripentol compared with fenfluramine and cannabidiol 20 mg/kg/day (Table 3).

**TABLE 3:** Indirect comparison of Discontinuations due to AEs for stiripentol versus fenfluramine versus cannabidiol.

Indirect comparisons—Risk differences [95% confidence interval] for discontinuations due to AEs								
A—using full cannabidiol trial populations								
	STP	*p*‐value	Placebo	*p*‐value	CBD10	*p*‐value	FFA0_7	*p*‐value
STP	STP							
Placebo	−0.13 [−0.30; 0.03]	0.12	Placebo					
CBD10	−0.14 [−0.30; 0.03]	0.09	−0.00 [−0.04; 0.03]	1	CBD10			
FFA0_7	**−0.18 [−0.36; −0.01]**	**0.0434**	−0.05 [−0.11; 0.01]	0.10	−0.05 [−0.12; 0.02]	0.16	FFA0_7	
CBD20	**−0.22 [−0.39; −0.05]**	**0.0112**	**−0.09 [−0.15; −0.03**]	**0.0033**	**−0.09 [−0.15; −0.03]**	**0.0033**	−0.04 [−0.12; 0.04]	0.33

B—using subgroup of cannabidiol trial populations taking clobazam								
	STP	*p*‐value	Placebo	*p*‐value	CBD10_CLB	*p*‐value	FFA0_7	*p*‐value
STP	STP							
Placebo	−0.13 [−0.29; 0.03]	0.11	Placebo					
CBD10_CLB	−0.14 [−0.30; 0.03]	0.09	−0.00 [−0.05; 0.04]	1	CBD10_CLB			
FFA0_7	**−0.18 [−0.35; −0.01]**	**0.0376**	−0.05 [−0.11; 0.01]	0.10	−0.05 [−0.12; 0.02]	0.16	FFA0_7	
CBD20_CLB	**−0.24 [−0.42; −0.06]**	**0.0090**	**−0.11 [−0.18; −0.03]**	**0.0041**	**−0.11 [−0.19; −0.03]**	**0.0071**	−0.06 [−0.15; 0.04]	0.22

*Note*: Treatments are ranked from best to worst along the leading diagonal. Indirect estimates of risk differences presented for treatments in columns versus treatments in rows. Bold figures are statistically significant (*p*‐value ≤0.05).

Abbreviations: CBD10, cannabidiol 10 mg/kg/day; CBD10_CLB, cannabidiol 10 mg/kg/day + clobazam; CBD20, cannabidiol 20 mg/kg/day; CBD20_CLB, cannabidiol 20 mg/kg/day + clobazam; FFA0_7, fenfluramine 0.7 mg/kg/day; STP, stiripentol 50 mg/kg/day.

### 3.4 Sensitivity analyses

Sensitivity analyses around the primary efficacy metric (≥50% reduction from baseline in MCSF) using the full trial populations demonstrate that the results are robust to the use of alternative models and measures of effect (Table S6). When restricting the analyses to fully published trials (i.e., excluding the STICLO‐Italy RCT of stiripentol and Study 3 RCT of fenfluramine, which are currently at the time of conductiong the analyses were only published in abstract or poster form) the efficacy of stiripentol remained statistically significantly superior to that of the cannabidiol regimens, and numerically superior to that with fenfluramine (Table S7). Results from the full trial network are, therefore, not subject to a high degree of uncertainty due to the inclusion of RCTs that are not fully published. Furthermore, results are consistent with expectations from the individual trial data, which show consistent treatment effects, and there is no evidence of inconsistencies between the direct and indirect evidence in the network. The results are, therefore, sufficiently robust to draw conclusions on the relative treatment effects of stiripentol, fenfluramine, and cannabidiol.

## 4 DISCUSSION

We assessed the comparative efficacy and safety of stiripentol, fenfluramine, and cannabidiol, as first‐line add‐on therapies, across all their licensed dose regimens in DS. Using available RCT data in robust NMAs, stiripentol, and fenfluramine had similar efficacy in achieving ≥50% (clinically meaningful) and ≥75% (profound) reductions from baseline in MCSF, and both were statistically superior to all licensed dose regimens of cannabidiol for these outcomes. Stiripentol was statistically superior to both fenfluramine and all licensed dose regimens of cannabidiol for achieving 100% reduction from baseline in MCSF. There were no statistically significant differences in the proportions of patients experiencing SAEs, but the risk of discontinuations due to AEs was lower for stiripentol compared with fenfluramine and cannabidiol 20 mg/kg/day. These results support the recommendations of the international consensus paper that positions stiripentol and fenfluramine ahead of cannabidiol in the DS treatment pathway.^9^

Three other NMA studies have been published and reached similar conclusions on the relative efficacy of stiripentol, fenfluramine, and cannabidiol in DS.^39^, ^40^, ^41^ However, these studies did not consider the efficacy of cannabidiol specifically in combination with clobazam, per its European licensed indication,^17^ and merged treatment effects across RCTs conducted in different lines of therapy, resulting in arguably medically illogical comparisons of data for fenfluramine plus stiripentol versus stiripentol alone. Two of these studies^39^, ^40^ incorrectly compared the incidence of *severe* adverse events with stiripentol against the incidence of *serious* adverse events with fenfluramine and cannabidiol, leading to erroneous conclusions on the relative safety of the add‐on therapies.^42^ Another reported NMA, conducted by two of the current authors, excluded stiripentol.^43^ Using correct data comparisons and further meaningful efficacy and safety outcome measures, the current study provides an up‐to‐date, robust assessment of the relative efficacy and safety of these three treatments as first‐line add‐on therapies across their licensed dose regimens, based on their available RCT data.

### 4.1 Limitations

There are some limitations to the data available for use in our analyses, and those of previously published NMAs, that must be acknowledged. DS is a rare disease and the RCTs included in these NMAs are relatively small. However, given the observed effect sizes across a range of outcome measures and in sensitivity and scenario analyses, the results of our NMAs are consistent in finding that, on average, stiripentol and fenfluramine provide superior seizure reductions versus all licensed dose regimens of cannabidiol when used as first‐line add‐on therapies.

The RCTs are of limited duration, providing 8–14 weeks of comparative treatment. Notably, the recruitment of patients to the STICLO‐France study of stiripentol was terminated prematurely by the independent data monitoring board due to the profound treatment benefit observed over placebo at an interim analysis.^44^, ^45^ It would be unethical to maintain patients on placebo for longer periods. The timing of endpoint assessment also differed between the stiripentol RCTs (last 4 weeks of the 8‐week treatment duration), and the fenfluramine and cannabidiol trials (throughout the 14‐week treatment duration). It is not possible to adjust for these differences; however, several observational studies have reported treatment effects with stiripentol that were well maintained over substantially longer timeframes than the RCTs^46^, ^47^, ^48^, ^49^ and so it is plausible that the results of the NMA could be applicable over the long term.

As the stiripentol trials were initiated 15–20 years before the fenfluramine and cannabidiol trials, it is possible that the approach to patient management may have differed; however, the valproate‐ and clobazam‐based standard of care therapy in the stiripentol trials reflects current treatment recommendations,^9^, ^10^ and the trials were accepted as appropriate by the US Food and Drug Administration agency for the licensing of stiripentol in 2018.^14^ Concomitant ASMs are potential effect modifiers, and heterogeneity in these could influence the NMA results. Only the STICLO trials of stiripentol were homogenous for concomitant ASMs, with all enrolled patients required to take valproate and clobazam. In contrast, the fenfluramine and cannabidiol RCTs permitted a broad range of concomitant ASMs. Concomitant clobazam is mandated in stiripentol licenses across the globe, but a high proportion of patients in the fenfluramine and cannabidiol RCTs were not taking clobazam concomitantly. Clobazam is not a significant effect modifier of fenfluramine,^15^ but is a significant effect modifier of cannabidiol, as reflected in cannabidiol's European license.^17^ In contrast to three other published NMAs,^39^, ^40^, ^41^ we conducted separate NMAs to ensure treatment effects were captured for both the overall cannabidiol trial populations (irrespective of concomitant clobazam use) and the subgroup of patients taking cannabidiol with concomitant clobazam (per its European license).

Stiripentol is a significant effect modifier for fenfluramine.^14^, ^15^ Fenfluramine Study 2^21^ was excluded from the NMAs because all enrolled patients were required to take concomitant stiripentol, which precludes a comparison against stiripentol, particularly in the context of first‐line add‐on use. The fenfluramine data for the NMAs are therefore taken from Study 1 and Study 3 in which no patients were taking concomitant stiripentol. Although around half of patients enrolled in Study 1 had prior experience with stiripentol, the efficacy of fenfluramine was similar in these patients as in the whole trial population.^50^ It is therefore reasonable to include data from the whole of the Study 1 and Study 3 populations in the NMAs.

Significant proportions of participants (33%–49%) in the cannabidiol trials were receiving concomitant stiripentol.^22^, ^23^ As there are no publicly available data for cannabidiol specifically in patients not taking stiripentol, it was necessary to adopt in all the NMAs the data from the cannabidiol trial populations irrespective of whether they were taking concomitant stiripentol. As regulatory analyses of the GWPCARE1 (part B) trial indicate that the reduction in seizure frequency with cannabidiol plus stiripentol is marginally greater than in the whole trial population,^51^ the adoption of the whole trial population in the NMAs to represent patients treated with cannabidiol without concomitant stiripentol is potentially conservative.

Efficacy comparisons were limited to convulsive seizure reduction responder rates (i.e., the proportion of patients achieving ≥50%, ≥75%, and 100% reductions from baseline in MCSF). It was not possible to conduct NMAs for the absolute change from baseline in MCSF for all three interventions due to differential reporting. Furthermore, SE and SUDEP events occur too infrequently in the DS trial setting, and non‐convulsive seizure reporting is unlikely to be as reliable as convulsive seizure reporting for comparative purposes. Nonetheless, convulsive seizure reduction responder rates are used consistently across the RCTs and by regulatory authorities and reflect key aims of ASM therapy in DS.

Finally, while the results of the NMAs are applicable on average, there are clinical factors beyond those considered in the NMA that would influence the choice of treatment for an individual patient. For example, in the USA, stiripentol is licensed for use in DS from age 6 months,^12^ whereas cannabidiol is from age 1 year^16^ and fenfluramine from age 2 years.^14^ While all three agents are associated with AEs of somnolence/sedation (which may be managed by dose modification of each agent or the concomitant ASMs), and gastrointestinal disturbance, loss of appetite, and weight loss,^12^, ^13^, ^14^, ^15^, ^16^, ^17^ there are additional, agent‐specific AE risks to consider: stiripentol is associated with a risk of neutropenia and thrombocytopenia when taken with valproate and clobazam^12^, ^13^; fenfluramine carries a boxed warning and risk evaluation and mitigation strategy (REMS) program in the USA, and risk management program in Europe, for valvular heart disease and pulmonary arterial hypertension^14^, ^15^; and, cannabidiol is associated with a risk of hepatocellular injury, particularly when used with valproate.^16^, ^17^ The potential for pharmacokinetic drug interactions within and between the add‐on therapies and other ASMs should also be considered, per their individual product labels.^12^, ^13^, ^14^, ^15^, ^16^, ^17^

## 5 CONCLUSIONS

NMAs using RCT data indicate stiripentol, as a first‐line add‐on therapy in DS, is at least as effective as fenfluramine and both are more effective than cannabidiol in reducing convulsive seizures. No significant difference in the incidence of SAEs between the three add‐on agents was observed, but stiripentol may have a lower risk of discontinuations due to AEs. Despite some data limitations, the results appear to be reliable, support international consensus‐based recommendations that position stiripentol and fenfluramine ahead of cannabidiol in the DS treatment pathway,^9^ and may inform clinical decision‐making and the continued development of guidelines.

## AUTHOR CONTRIBUTIONS

TT and DV conceived the study. TT and WL conducted the systematic review, analyses, and manuscript drafting. All authors critically reviewed the manuscript for intellectual content and integrated revisions. RG and CC contributed equally to this study. All authors agreed to the publication of the manuscript.

## FUNDING INFORMATION

This study was funded by Biocodex, the manufacturer of DIACOMIT® (stiripentol).

## CONFLICT OF INTEREST STATEMENT

RG received fees for Advisory Boards from UCB, Zogenix, Biocodex, GW‐Jazz, Angelini, Takeda, and Rapport Therapeutics, and was an investigator in the STICLO‐Italy trial of stiripentol. CC received fees for Advisory Boards from Advicenne, Zogenix, Neuren, Biocodex, EISAI, GW‐Jazz, BIAL, and Orphelia, and was an investigator in the STICLO‐France trial of stiripentol. DV is an employee of Biocodex, the manufacturer of stiripentol. TT is a past employee of Zogenix International Ltd/UCB, is Director of Henley Health Economics Ltd and received consulting fees from Biocodex for this and other projects. WL is a past employee of Zogenix International Ltd/UCB, is Director of Paragon Market Access Ltd and received consulting fees from Henley Health Economics Ltd for this and other projects. All authors abided by their ongoing confidentiality and contractual obligations.

## ETHICS STATEMENT

We the authors confirm that we have read the Journal's position on issues involved in ethical publication and affirm that this report is consistent with those guidelines.

## Supporting information


Data S1

## Data Availability

All data used in the analyses are provided in the manuscript tables.
