# Supplementary material for: Comparative efficacy and safety of stiripentol, cannabidiol and fenfluramine as first‐line add‐on therapies for seizures in Dravet syndrome: A network meta‐analysis
Source: Epilepsia Open. 2024 Mar 1;9(2):689–703. doi: 10.1002/epi4.12923 (PMC10984299; doi:10.1002/epi4.12923)
Supplement: Supplementary file 1 — Data S1 [file EPI4-9-689-s001.docx]

**Comparative efficacy and safety of stiripentol, cannabidiol and fenfluramine as first-line add-on therapies for seizures in Dravet syndrome: a network meta-analysis study – Supplementary Information**

**Table S 1. Search strategy sample (PubMed)**

| **PubMed Search strategy (to 30/06/2023)** | **Returned hits** |
| --- | --- |
| #1 “Epilepsies, Myoclonic”[Mesh] | 5425 |
| #2 (child OR childhood OR children OR infan*) | 3737872 |
| #3 #1 AND #2 | 2693 |
| #4 “dravet syndrome” | 1454 |
| #5 “severe myoclonic epilepsy” | 386 |
| #6 SMEI | 182 |
| #7 Dravet* | 1804 |
| #8 "dravet's syndrome" | 41 |
| #9 “childhood epileptic encephalopathy” | 35 |
| #10 “childhood epilepsy encephalopathy” | 7559 |
| #11 “childhood epileptic encephalopathies” | 23 |
| #12 “childhood epilepsy encephalopathies” | 7559 |
| #13 #3 OR #4 OR #5 OR #6 OR #7 OR #8 OR #9 OR #10 OR #11 OR #12 | 10775 |
| #14 randomized controlled trial [pt] | 597342 |
| #15 controlled clinical trial [pt] | 687790 |
| #16 randomized [tiab] | 665153 |
| #17 placebo [tiab] | 246561 |
| #18 clinical trials as topic [mesh: noexp] | 383034 |
| #19 randomly [tiab] | 412804 |
| #20 trial [ti] | 288368 |
| #21 #14 OR #15 OR #16 OR #17 OR #18 OR #19 OR #20 | 1629324 |
| #22 #13 AND #21 | 547 |
| #23 animals [mh] NOT humans [mh] | 5135662 |
| #24 #22 NOT #23 | **551** |
| Dravet syndrome search terms informed by searches conducted in NICE TA614, 2018 (see: <https://www.nice.org.uk/guidance/ta614/evidence/appraisal-consultation-committee-papers-pdf-7017624830>) | |

**Table S 2. Eligibility criteria**

| **Inclusion criteria** | |
| --- | --- |
| Population | - Patients of any age with a diagnosis of DS (with or without confirmed *SCN1A* mutation). - If studies include mixed patient populations, only those reporting results separately for DS patients to be included |
| Intervention(s) | - Stiripentol licensed doses of up to 50mg/kg/day - Fenfluramine licensed doses of 0.7 mg/kg/day, or 0.4 mg/kg/day if co-administered with stiripentol (including fenfluramine expressed as the hydrochloride salt at doses of 0.8mg/kg/day, or 0.5 mg/kg/day if co-administered with stiripentol. - Pharmaceutical grade cannabidiolasused in the Epidyolex/Epidiolex formulation, at licensed doses of up to 10mg/kg/day or 20mg/kg/day |
| Comparator(s) | - Any drug comparator used as add-on therapy to standard of care ASMs - Placebo/standard of care ASMs |
| Outcomes | - Any outcome relating to: - Change in convulsive seizure frequency from baseline - Responder rates (>50% [clinically meaningful], >75% [profound] or 100% reduction from baseline in convulsive seizure frequency) - Serious adverse events rates - Discontinuations due to adverse events |
| Study designs | - Any RCTs involving at least 10 subjects per treatment arm – including re-analyses of RCTs - HTA and regulatory reports providing RCT data - Systematic literature reviews – for background information, reference checking and any additional RCT data not provided in primary publications |
| Location, setting, and language | - Any geographic location or setting - Any language |

**Table S 3. Study designs, eligibility criteria and endpoint assessments**

| **Design element** | **Study** | | | | | | | | |
| --- | --- | --- | --- | --- | --- | --- | --- | --- | --- |
|  | **Stiripentol**  **STICLO-France** (1) | **Stiripentol**  **STICLO-Italy** (2) | **Fenfluramine**  **Study 1** (3) | **Fenfluramine**  **Study 2** (4) | **Fenfluramine**  **Study 3** (5) | **Cannabidiol GWPCARE1 Part A** (6) | **Cannabidiol**  **GWPCARE1 Part B** (7) | **Cannabidiol**  **GWPCARE2** (8) | |
| **Intervention and**  **comparator** | STP 50mg/kg/day or placebo | STP 50mg/kg/day or placebo | FFA 0.7mg/kg/day (max. 26mg/day) or  FFA 0.2mg/kg/day or  placebo | FFA 0.4mg/kg/day (max. 17mg/day) or  placebo | FFA 0.7mg/kg/day (max. 26mg/day) or  FFA 0.2mg/kg/day or  placebo | CBD 5mg/kg/day or CBD 10mg/kg/day or CBD 20mg/kg/day or placebo | CBD 20mg/kg/day or placebo | CBD 10mg/kg/day or CBD 20mg/kg/day or placebo | |
| **Study design** | Phase 3 Placebo-controlled RCT | | Phase 3 placebo-controlled RCT | | | Placebo-controlled RCT | Phase 3 placebo controlled RCT | | |
| **n** | 41 | 23 | 119 | 87 | 143 | 34 | 120 in full trial  (78 in subgroup taking CLB) | 198 in full trial  (126 in subgroup taking CLB) | |
| **Year(s) conducted** | 1996-1998 | 1999-2000 | 2016–2018 | 2016–2018 | 2016-2020 | 2014-2015 | 2015 | 2015–2018 | |
| **Study and treatment duration** | 1 month baseline, 2 months treatment | | 6-week baseline, 2- week titration + 12- week maintenance | 6-week baseline, 3- week titration + 12- week maintenance | 6-week baseline, 2- week titration + 12- week maintenance | 4-week baseline, 3-week treatment (including titration) | 4-week baseline, 2- week titration + 12- week maintenance | |  |
| **Eligibility** | - Dravet Syndrome - 3–18 years old - ≥4 generalised clonic or tonic-clonic seizures per month during baseline | | - Dravet Syndrome - 2–18 years old - ≥4 convulsive seizures per 4- week period during previous 12 weeks prior to screening | | - Dravet Syndrome - 2–18 years old | - Dravet Syndrome - 4-10 years old - <4 convulsive seizures during 4 week baseline | - Dravet Syndrome - 2–18 years old   ≥4 convulsive seizures during 28-day baseline | |  |
| **Background ASM** | - 100% receiving VPA + CLB | | - One or more stable ASMs - All other medications or interventions must be stable for ≥4 weeks prior to screening and are expected to remain stable throughout the study - No STP in the 21 days prior to screening | - Receiving stable dose of CLB, and/or VPA, and 100% STP - All medications or interventions for epilepsy must be stable for ≥4 weeks prior to screening and are expected to remain stable throughout the study | - One or more stable ASMs - No STP in the 21 days prior to screening | - One or more stable ASMs | - One or more stable ASMs - All medications or interventions must be stable for ≥4 weeks prior to screening and are expected to remain stable throughout the study | |  |
| **Convulsive seizure definition** | Generalised clonic or tonic-clonic | | Generalised tonic-clonic, tonic, clonic, tonic-atonic, hemiclonic, and focal seizures with an observable motor component | | NS | Tonic, clonic, tonic-clonic or atonic | | |  |
| **Reduction in convulsive seizures reporting** | % change from baseline in MCSF after 1^st^ month and after 2^nd^ month of treatment period compared with baseline  (secondary endpoint)  Mean % reductions provided for each arm | | % change in MCSF between baseline and T+M periods (per 28 days)  (primary endpoint)  Parametric assessment of % reduction from baseline in convulsive seizure frequency per 28 days compared with placebo  Median % reductions provided | | | NR | % change in MCSF between baseline and T+M periods (per 28 days)  (primary endpoint)  Parametric assessment of % reduction from baseline in convulsive seizure frequency per 28 days compared with placebo  Median % reductions provided | |  |
| **Responder rate (>50, >75 and 100% reduction from baseline in MCSF) reporting** | Reported MCSF for 2^nd^ month of treatment period compared with baseline (per 30 days)  (50% responder rate was primary endpoint) | | Reported MCSF over combined T+M period (per 28 days) vs baseline  (key secondary endpoints) | | | NS | Reported MCSF over combined T+M period (per 28 days)  (key secondary endpoint) | |  |
| **Any AE reporting** | Rates reported over treatment duration | NS | Rates reported over treatment duration | | | Rates reported over treatment duration | Rates reported over treatment duration | |  |
| **SAE reporting** | Rates reported over treatment duration (NB: reported in regulatory documentation) | |  |  |  | NS |  |  |  |
| **Discontinuations due to AE reporting** |  |  |  |  |  |  |  |  |  |
| **Key:** AE, adverse events, ASM, anti-seizure medication; CBD, cannabidiol; CLB, clobazam; MCSF, monthly convulsive seizure frequency; FFA, fenfluramine; NB, Nota Bene (please note); NR, not reported; NS, not stated in available publication; RCT, randomized controlled trial; SAE, serious AE; STP, stiripentol; T+M, titration and maintenance treatment period; VPA, valproate | | | | | | | | |  |

**Table S 4. Baseline characteristics of study participants (excluding GWPCARE1 Part A)**

| **Characteristics** | **Study** | | | | | | | | |
| --- | --- | --- | --- | --- | --- | --- | --- | --- | --- |
|  | **Stiripentol**  **STICLO-France** (1) | **Stiripentol STICLO-Italy**  (2) | **Fenfluramine**  **Study 1** (3) | **Fenfluramine Study 2** (4) | **Fenfluramine Study 3** (5) | **Cannabidiol GWPCARE1 Part B (full trial population)** (7) | **Cannabidiol GWPCARE2 (full trial population)** (8) | **Cannabidiol 10mg/kg/day + Clobazam subgroup** (9) | **Cannabidiol 20mg/kg/day + Clobazam subgroup** (9) |
| **N** | 41 | 23 | 119 | 87 | 143 | 120 | 198 | 45 | 80 |
| **Age – yrs (mean±SD)** | ~9.4 | 9.1 + 4 | 9.0 ±4.65 | 9.1 ±4.80 | ~9 | 9.8 ±4.8 | 9.3 ±4.4 | 9.1+4.1 | 9.3+4.3 |
| **Male – n (%)** | 17 (41.5) | 13 (56.5) | 64 (54) | 50 (57.5) | 74 (51.7) | 62 (52) | 94 (47.5) | 23 (51) | 45 (56) |
| **BMI – kg/m^2^ (mean±SD)** | NR | NR | 18.57 ±4.408 | 18.24 ±4.049 | NR | 18.7±4.6 | 18.7±4.3 | 18.9 | 19.0 |
| **White** | NR | NR | 98 (82.4) | 52 (59.8) | 106 (74.1) | 94 (78.3) | 176 (88.9) | NR | NR |
| **Black or African American** | NR | NR | NR | 3 (3.4) | NR | 4 (3.3) | 5 (2.5) | NR | NR |
| **Asian** | NR | NR | 7 (5.9) | 3 (3.4) | 20 (14.0) | 1 (0.8) | 5 (2.5) | NR | NR |
| **Other/not reported/unknown** | 41 (100) | 23 (100) | 14 (11.8) | 29 (33.3) | 16 (11.2) | 21 (17.5) | 12 (6.0) | 45 (100) | 80 (100) |
| **USA** | 0 | 0 | 70 (58.8) | 22 (25.3) | NR | 72 (60) | 93 (47) | 22 (49) | 44 (55) |
| **Rest of world** | 41 (100) | 23 (100) | 49 (41.2) | 65 (74.7) | NR | 48 (40) | 105 (53) | 23 (51) | 36 (45) |
| **Baseline MCSF** | Per 30 days:  STP 50mg/kg/day: median 18  (IQR 4-73)  Placebo: 19  (IQR 4-76) | Per 30 days:  STP 50mg/kg/day: mean 27.4±28.6  Placebo: 33.6±28.2 | Per 28 days:  FFA 0.2 mg/kg/day: median 17.5  (range 4.7– 623.5)  FFA 0.7 mg/kg/day: median 20.7 (range 4.8–124.0)  Placebo:  median 27.3  (range 3.3–147.3) | Per 28 days:  FFA 0.4 mg/kg/day: median 14.0  (range 2.7– 213.3)  Placebo:  median 10.7  (range 2.7–162.7) | Per 28 days:  FFA 0.2 mg/kg/day: median 18.0  (range 4.0– 1464)  FFA 0.7 mg/kg/day: median 13.0 (range 2.7–2701)  Placebo:  median 12.7  (range 4.0–229.3) | Per 28 days:  CBD 20 mg/kg/day: median 12.4  (range 3.9–1717)  Placebo:  median 14.9  (range 3.7–718) | Per 28 days:  CBD 20  mg/kg/day:  median 9.0  (range 3.9–661.2)  (IQR 6, 21)  CBD 10  mg/kg/day:  median 13.5  (range 0–467.0)  (IQR 6, 31)  Placebo:  median 16.6  (range 3.0–770.5)  (IQR 7, 51) | Per 28 days:  GWPCARE1B:  CBD 20 mg/kg/day: median 9.6  (range 3.9–661.2)  GWPCARE2:  CBD 10 mg/kg/day: median 13.1  (range 4.0–238.4)  CBD 20 mg/kg/day: median 10.8  (range 3.9–553,5)  Placebo (n=79):  median 17.0  (range 3.0–448.9) | |
| **Previous ASMs** | NR | NR | NR | NR | NR | 4.6±3.8 | Median 4 | Median 4 | Median 4 |
| **Concomitant ASMs** | Mean 2.2 | NR | Mean 2.4 | Mean 3.5 | NR | Mean 2.9±1.0 | Median 3 | Median 3 | Median 3 |
| **Clobazam** | 41 (100) | 23 (100) | 70 (58.8) | 82 (94.3) | NR | 78 (65) | 126 (64) | 45 (100) | 80 (100) |
| **Valproate, all forms** | 41 (100) | 23 (100) | 71 (59.7) | 66 (75.9) | NR | 71 (59) | 139 (70) | 30 (67) | 56 (70) |
| **Stiripentol** | - | - | 0 | 87 (100) | NR | 51 (42) | 71 (36) | 17 (38) | 51 (51) |
| **Levetiracetam** | 0 | 0 | 26 (21.8) | 10 (11.5) | NR | 33 (28) | 54 (27) | 11 (24) | 15 (19) |
| **Topiramate** | 0 | 0 | 30 (25.2) | 21 (24.1) | NR | 31 (26) | 46 (23) | 8 (18) | 18 (23) |
| **Key:** ASM; antiseizure medication; BMI, body mass index; CBD, cannabidiol; FFA, fenfluramine; NR, not reported; IQR, interquartile range; MCSF, monthly convulsive seizure frequency; No., number; SD, standard deviation; STP, stiripentol; yrs, years | | | | | | | | | |

**Table S 5.** **Indirect comparison of SAEs for stiripentol vs fenfluramine vs cannabidiol**

| **Indirect comparisons – Risk Differences [95% Confidence Interval] for experiencing SAEs** | | | | | | | | |
| --- | --- | --- | --- | --- | --- | --- | --- | --- |
| **A – using full cannabidiol trial populations** | | | | | | | | |
|  | STP | p-value | Placebo | p-value | FFA0_7 | p-value | CBD10 | p-value |
| STP | STP |  |  |  | . |  | . |  |
| Placebo | -0.05  [-0.26; 0.15] | 0.65 | Placebo |  |  |  |  |  |
| FFA0_7 | -0.08  [-0.29; 0.14] | 0.48 | -0.02  [-0.10; 0.05] | 0.61 | FFA0_7 |  | . |  |
| CBD10 | -0.10  [-0.34; 0.13] | 0.41 | -0.05  [-0.17; 0.08] | 0.44 | -0.03  [-0.17; 0.12] | 0.70 | CBD10 |  |
| CBD20 | -0.16  [-0.38; 0.06] | 0.15 | **-0.11**  **[-0.19; -0.02]** | **0.0112** | -0.09  [-0.20; 0.03] | 0.13 | -0.06  [-0.19; 0.07] | 0.37 |
| **B – using subgroup of cannabidiol trial populations taking clobazam** | | | | | | | | |
|  | STP | p-value | Placebo | p-value | FFA0_7 | p-value | CBD10_CLB | p-value |
| STP | STP |  |  |  | . |  | . |  |
| Placebo | -0.05  [-0.26; 0.15] | 0.65 | Placebo |  |  |  |  |  |
| FFA0_7 | -0.08  [-0.29; 0.14] | 0.48 | -0.02  [-0.10; 0.05] | 0.61 | FFA0_7 |  | . |  |
| CBD10_CLB | -0.13  [-0.39; 0.12] | 0.32 | -0.08  [-0.24; 0.08] | 0.33 | -0.06  [-0.23; 0.12] | 0.51 | CBD10_CLB |  |
| CBD20_CLB | -0.20  [-0.43; 0.03] | 0.09 | **-0.15**  **[-0.25; -0.04]** | **0.0051** | -0.13  [-0.26; 0.01] | 0.0587 | -0.07  [-0.23; 0.09] | 0.40 |
| Treatments are ranked from best to worst along the leading diagonal. Indirect estimates of risk differences presented for treatments in columns vs treatments in rows. Bold figures are statistically significant (p-value <0.05).  CBD10, cannabidiol 10mg/kg/day; CBD10_CLB, cannabidiol 10mg/kg/day + clobazam; CBD20, cannabidiol 20mg/kg/day; CBD20_CLB, cannabidiol 20mg/kg/day + clobazam; FFA0_7, fenfluramine 0.7mg/kg/day; STP, stiripentol 50mg/kg/day | | | | | | | | |

**Table S 6.** **Sensitivity analyses around 50% responder rate outcome, full trial populations**

| **Sensitivity analyses – 50% responder rates [95% Confidence Interval] utilising full cannabidiol trial populations** | | | | | | |
| --- | --- | --- | --- | --- | --- | --- |
|  | **Random effects models** | | | **Fixed effect models** | | |
|  | **RR** | **OR** | **RD** | **RR** | **OR** | **RD** |
| STP vs  placebo | 10.20  [2.62; 39.66] | 31.75  [6.25; 161.16] | 0.64  [0.46; 0.81] | 10.20  [2.62; 39.66] | 31.75  [6.25; 161.16] | 0.64  [0.46; 0.81] |
| FFA0_7 vs placebo | 7.20  [3.67; 14.11] | 22.75  [9.55; 54.23] | 0.62  [0.51; 0.74] | 7.20  [3.67; 14.11] | 22.75  [9.55; 54.23] | 0.62  [0.51; 0.74] |
| CBD10 vs placebo | 1.58 [1.03; 2.42] | 2.03  [1.03; 4.00] | 0.16  [0.01; 0.13] | 1.58  [1.03; 2.42] | 2.03  [1.03; 4.00] | 0.16  [0.01; 0.13] |
| CBD20 vs placebo | 1.73 [1.22; 2.45] | 2.36  [1.39; 4.00] | 0.19  [0.08; 0.31] | 1.73  [1.22; 2.45] | 2.36  [1.39; 4.00] | 0.19  [0.08; 0.31] |
| CBD10, cannabidiol 10mg/kg/day; CBD20, cannabidiol 20mg/kg/day; FFA0_7, fenfluramine 0.7mg/kg/day; OR, odds ratio; RD, risk difference; RR, relative risk; STP, stiripentol 50mg/kg/day | | | | | | |

**Table S 7.** **Scenario analysis: Indirect comparisons for 50% responder rates using only fully published, full trial populations**

| **Indirect comparisons – Risk Differences [95% Confidence Interval] for 50% responder rates** | | | | | | | | |
| --- | --- | --- | --- | --- | --- | --- | --- | --- |
| **Using fully published, full trial populations** | | | | | | | | |
|  | STP | p-value | FFA0_7 | p-value | CBD20 | p-value | CBD10 | p-value |
| STP | STP |  | . |  | . |  | . |  |
| FFA0_7 | 0.11  [-0.17; 0.39] | 0.45 | FFA0_7 |  | . |  | . |  |
| CBD20 | **0.47**  **[ 0.22; 0.71]** | **0.0002** | **0.36**  **[ 0.14; 0.57]** | **0.0011** | CBD20 |  |  |  |
| CBD10 | **0.50**  **[ 0.24; 0.77]** | **0.0001** | **0.39**  **[ 0.15; 0.62]** | **0.0012** | 0.03  [-0.12; 0.19] | 0.72 | CBD10 |  |
| Placebo | **0.66**  **[ 0.45; 0.88]** | **0.0002** | **0.55**  **[ 0.37; 0.73]** | **<0.0001** | **0.19**  **[ 0.08; 0.31]** | **0.0013** | **0.16**  **[ 0.01; 0.31]** | **0.0363** |
| Treatments are ranked from best to worst along the leading diagonal. Indirect estimates of risk differences presented for treatments in columns vs treatments in rows. Bold figures are statistically significant (p-value <0.05).  CBD10, cannabidiol 10mg/kg/day; CBD20, cannabidiol 20mg/kg/day; FFA0_7, fenfluramine 0.7mg/kg/day; STP, stiripentol 50mg/kg/day | | | | | | | | |

***Figure S 1. PRISMA flow diagram***


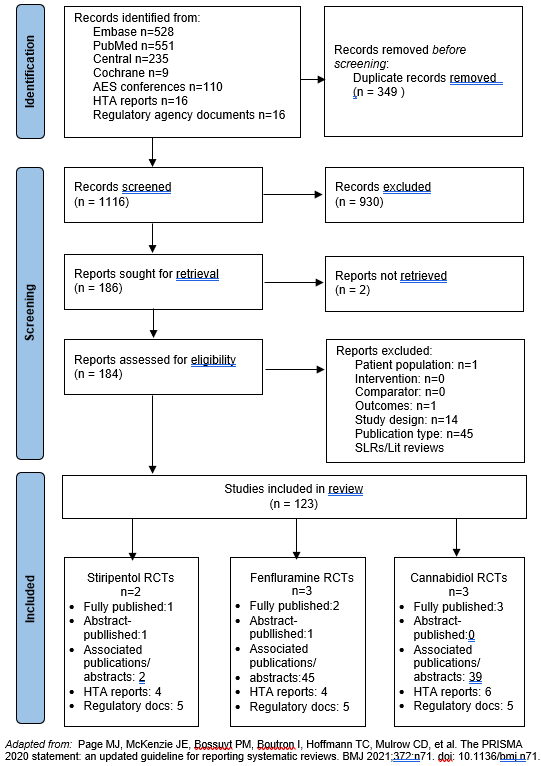


**Figure S 2. Risk of bias assessment using Cochrane Risk of Bias 2 Tool**


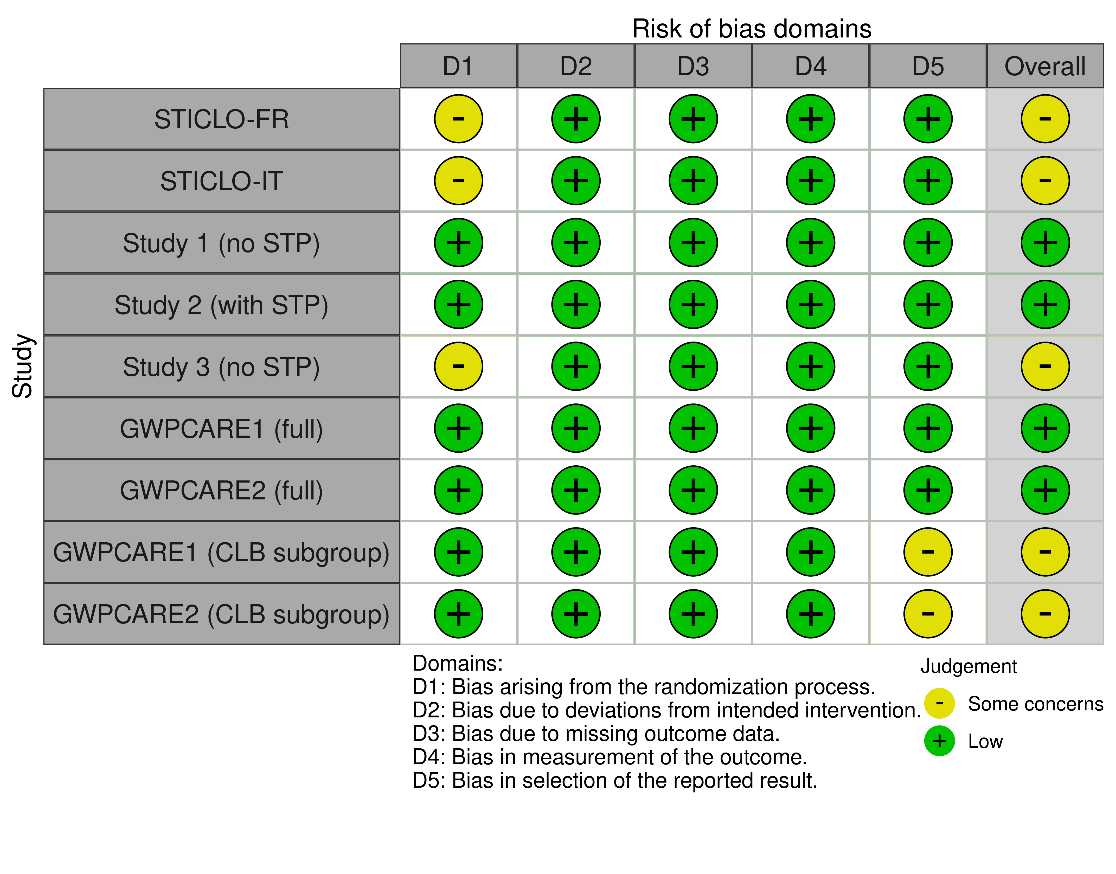


Generated using robvis: McGuinness, LA, Higgins, JPT. Risk-of-bias VISualization (robvis): An R package and Shiny web app for visualizing risk-of-bias assessments. Res Syn Meth. 2020; 1- 7. <https://doi.org/10.1002/jrsm.1411>. Available at: <https://www.riskofbias.info/welcome/robvis-visualization-tool>.

**Figure S 3. Pairwise relative risks of achieving >50% reduction in MCSF vs placebo**


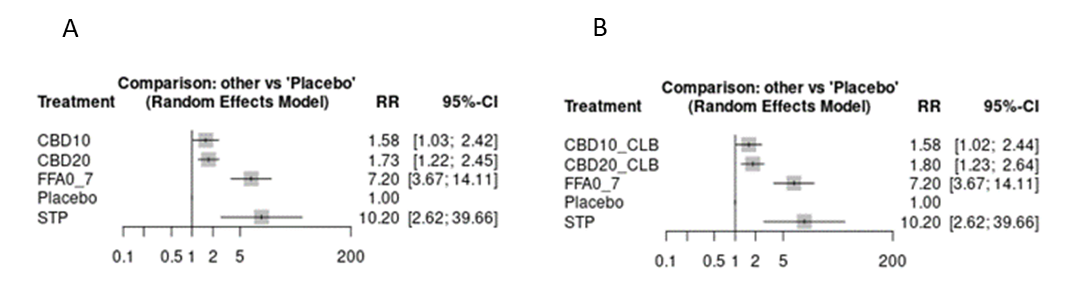


Key: A – 50% responder rates using full cannabidiol trial populations; B – 50% responder rates using subgroup of cannabidiol trial populations taking clobazam. CBD10, cannabidiol 10mg/kg/day; CBD10_CLB, cannabidiol 10mg/kg/day + clobazam; CBD20, cannabidiol 20mg/kg/day; CBD20_CLB, cannabidiol 20mg/kg/day + clobazam; FFA0_7, fenfluramine 0.7mg/kg/day; MCSF, monthly convulsive seizure frequency; RR, relative risk; STP, stiripentol 50mg/kg/day; 95%-CI, 95% confidence intervals

**Figure S 4. Pairwise relative risks of experiencing SAEs**


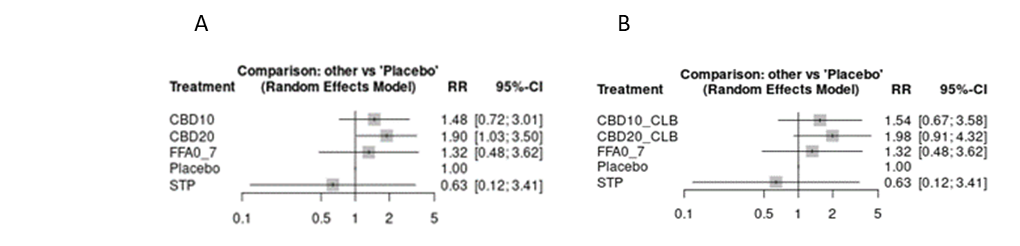


Key: A – SAE relative risks using full cannabidiol trial populations; B – SAE relative risks using subgroup of cannabidiol trial populations taking clobazam. CBD10, cannabidiol 10mg/kg/day; CBD10_CLB, cannabidiol 10mg/kg/day + clobazam; CBD20, cannabidiol 20mg/kg/day; CBD20_CLB, cannabidiol 20mg/kg/day + clobazam; FFA0_7, fenfluramine 0.7mg/kg/day; MCSF, monthly convulsive seizure frequency; RR, relative risk; STP, stiripentol 50mg/kg/day; 95%-CI, 95% confidence intervals.

***Supplementary Information References***

1. Chiron C, Marchand MC, Tran A, Rey E, d’Athis P, Vincent J, et al. Stiripentol in severe myoclonic epilepsy in infancy: a randomised placebo-controlled syndrome-dedicated trial. STICLO study group. Lancet (London, England). 2000/11/23 ed. 2000 Nov 11;356(9242):1638–42.

2. Guerrini R, Tonnelier S, d’Athis P, al. et. Stiripentol in severe myoclonic epilepsy in infancy (SMEI): a placebo-controlled Italian trial (P496). In Epilepsia; 2002.

3. Lagae L, Sullivan J, Knupp K, Laux L, Polster T, Nikanorova M, et al. Fenfluramine hydrochloride for the treatment of seizures in Dravet syndrome: a randomised, double-blind, placebo-controlled trial. Lancet. 2019;394(10216):2243–54.

4. Nabbout R, Mistry A, Zuberi S, Villeneuve N, Gil-Nagel A, Sanchez-Carpintero R, et al. Fenfluramine for Treatment-Resistant Seizures in Patients With Dravet Syndrome Receiving Stiripentol-Inclusive Regimens: a Randomized Clinical Trial. JAMA neurology. 2020;77(3):300‐308.

5. Sullivan J. 853. Fenfluramine (FINTEPLA) in Dravet syndrome: Results of a third randomized, placebo-controlled clinical trial (Study 3). In: Virtual American Epilepsy Society (AES) Annual Meeting. Virtual American Epilepsy Society (AES) Annual Meeting; 2020.

6. Devinsky O, Patel AD, Thiele EA, Wong MH, Appleton R, Harden CL, et al. Randomized, dose-ranging safety trial of cannabidiol in Dravet syndrome. Neurology. 2018/03/16 ed. 2018 Apr 3;90(14):e1204–11.

7. Devinsky O, Cross JH, Laux L, Marsh E, Miller I, Nabbout R, et al. Trial of Cannabidiol for Drug-Resistant Seizures in the Dravet Syndrome. The New England journal of medicine. 2017/05/26 ed. 2017 May 25;376(21):2011–20.

8. Miller I, Scheffer IE, Gunning B, Sanchez-Carpintero R, Gil-Nagel A, Perry MS, et al. Dose-Ranging Effect of Adjunctive Oral Cannabidiol vs Placebo on Convulsive Seizure Frequency in Dravet Syndrome: A Randomized Clinical Trial. JAMA Neurol. 2020;77(5):613–21.

9. Gunning B. Cannabidiol in conjunction with clobazam: analysis of four randomized controlled trials. Acta Neurol Scand. 2021;143:154–63.
